# Intercropping of wheat alleviates the adverse effects of phenolic acids on faba bean

**DOI:** 10.3389/fpls.2022.997768

**Published:** 2022-10-17

**Authors:** Yiran Zheng, Yuting Guo, Yu Li, Wenhao Yang, Yan Dong

**Affiliations:** College of Resources and Environment, Yunnan Agricultural University, Kunming, China

**Keywords:** peroxidation, antioxidant enzymes, ultrastructure, intercropping, faba bean (*Vicia faba* L)

## Abstract

After years of continuous cultivation of faba beans (*Vicia faba* L.), autotoxic substances accumulate in the soil, leading to a high incidence of *Fusarium oxysporum* (FOF) wilt. Faba bean–wheat intercropping is often used to alleviate these problems. The goal of this research was to explore the role of benzoic acid and cinnamic acid in promoting the occurrence of faba bean *Fusarium* wilt and the potential mechanism of faba bean–wheat intercropping to control the occurrence of this disease. We established a field experiment and a hydroponic experiment that involved the inoculation of FOF and the exploration of exogenous addition of cinnamic acid and benzoic acid at different concentrations, the effects on the degree of peroxidation, resistance system, and ultrastructure of faba bean roots. In addition, the antioxidative response of faba bean–wheat intercropping against the autotoxicity of benzoic acid and cinnamic acid was examined. In the field experiment, compared with monoculture, faba bean–wheat intercropping effectively controlled the occurrence of *Fusarium* wilt, significantly reduced the contents of H_2_O_2_ and O_2_^−^ in faba bean roots, increased the expression and activity of antioxidant enzymes superoxide dismutase (SOD) and catalase (CAT), maintained cell stability, and significantly reduced the contents of benzoic acid and cinnamic acid in faba bean rhizosphere. In the pot experiment, it was found that compared with the control, different concentrations of benzoic acid and cinnamic acid (50, 100, and 200 mg·L^−1^) significantly increased the content of H_2_O_2_ and O_2_^−^ in faba bean, decreased the activity and gene expression of antioxidant enzymes SOD and CAT, and damaged cell membrane structure. Furthermore, it promoted the occurrence of *Fusarium* wilt of faba bean. The faba bean–wheat intercropping alleviated the stress. Benzoic acid and cinnamic acid can increase the content of hydrogen peroxide and superoxide anions in faba bean plants, reduce the enzymatic activity and expression of antioxidant enzyme genes, damage the cell membrane structure, and promote the occurrence of faba bean *Fusarium* wilt. The faba bean–wheat intercropping can effectively alleviate the autotoxicity of benzoic acid and cinnamic acid and reduce the occurrence of faba bean *Fusarium* wilt.

## Introduction

As the world’s population continues to grow, so does the demand for food (Tan et al., 2021). Owing to the limited amount of arable land, the amount of newly added arable land is very small, and the long-term planting of the same crops on the same land has become the most common pattern in agriculture (Tilman et al., 2011; Alexandratos and Bruinsma, 2012). After many years of planting the same crops, a high incidence of soil-borne diseases can appear (Xiong et al., 2015). *Fusarium* wilt is one of the more serious soil-borne diseases of crops, which has been reported worldwide and seriously endangers the health of a variety of crop production (Zhou et al., 2018). *Fusarium oxysporum* (FOF) is the main pathogen causing crop blight, where FOF preferentially colonizes, invades, and passes through the root cortex, reaching the xylem and then spreading to the aboveground stem in the process of infecting crops with FOF at the junction of lateral roots and taproots (Niño-Sánchez et al., 2015), and through the root cortex, reaching xylem and then spreading to the aboveground stem (De Borba et al., 2017). When FOF colonizes the upper ground stem, it causes damage to the xylem duct and deepens the degree of peroxidation. Cell breakdown due to deepening peroxidation is the leading cause of eventual morbidity and death of host crops (Beckman, 1987). In this process, the self-toxicity of host plants helps pathogenic bacteria to be an important reason for their successful invasion and pathogenicity.

Autotoxicity refers to the direct or indirect toxic effects of certain plants on the same plant or other plants of the same species or the same family by releasing chemicals through leaching from the aboveground parts, root exudates, and plant residues (Tanha et al., 2017). These chemicals are called autotoxic substances (Wang et al., 2015). In the long-term monoculture process, plants enrich fungal pathogens in the rhizosphere by releasing autotoxins and providing fungal pathogens with the nutrients needed for growth and reproduction. For example, amino acids, sugars, and other substances secreted by peanut roots significantly promote the growth and reproduction of *Fusarium* spp. and increase the risk of peanut soil-borne diseases (Li et al., 2013). Benzoic acid secreted by peanut roots significantly increased the abundance of *Fusarium* spp. in the peanut rhizosphere, promoted the mycelial growth and spore production of *Fusarium* spp., and aggravated the occurrence of root rot (Liu et al., 2016). The phenolic acid component in the tissue rot solution of watermelon can significantly promote the growth and reproduction of FOF and increase the risk of wilt disease in watermelons (Wu et al., 2014). However, in addition to promoting the growth of pathogenic bacteria, the autotoxins released by plants can also accelerate the pathogenic process of pathogenic bacteria by reducing the antioxidant capacity of plants. The balance between plant antioxidant capacity and peroxide level is an important factor to ensure plant health (Hasanuzzaman et al., 2020a; Hasanuzzaman et al., 2020b). After being stressed, plants will activate their antioxidant system, improve their antioxidant capacity, and enhance their resistance by producing a large number of plant growth regulators (Zulfiqar and Ashraf, 2021a). Catalase (CAT) and superoxide dismutase (SOD) are important indicators of plant antioxidant capacity. They can help plants remove excess H_2_O_2_ and O_2_^−^ from their tissues, alleviate the peroxidation damage caused by pathogenic bacteria, and reduce CAT. SOD activity is one of the important reasons for autotoxins to promote disease occurrence (Lv et al., 2018). Wang et al. (2019) showed that the syringic acid, phthalic acid, benzoic acid, and vanillic acid secreted by strawberries increase the risk of strawberry anthracnose by inhibiting the activity of CAT and SOD in strawberry roots. The autotoxic substances secreted by melons can increase the risk of melon disease by inhibiting the expression of SOD in the root system of melon (Zhang et al., 2018). In addition, the autotoxins will also damage the cellular structure by promoting oxidative bursts, resulting in disease and death of the plant (Zulfiqar and Ma, 2021b). Research by Yang et al. (2018) also proved that autotoxic substances significantly reduce the expression of genes related to cell membrane stability, destroy the structure of cell membranes, and ultimately damage cells and render the plants susceptible to disease. However, the existence of pathogens in actual production is a prerequisite for the disease of crops. These studies have not explored the pathogenic mechanism caused by the synergistic effect of autotoxic substances and pathogens.

Currently, chemical and physical methods have been widely used to control the occurrence of soil-borne diseases. Chemical methods include spraying fungicides (Fravel et al., 2005) or using the fumigant methyl bromide (Cebolla et al., 2000), while physical methods include heat-covered film insulation (Bonanomi et al., 2008). However, these methods are not environmentally friendly, are often limited by unstable effects under field conditions, and cannot provide consistent and reliable results (Lv et al., 2018). Intercropping is an internationally recognized planting mode that can effectively increase crop yields and prevent pests and diseases (Gebru, 2015). Intercropping has played a significant role, particularly in the control of soil-borne diseases (Lopes et al., 2016). The intercropping of marigold and tomato inhibited the damage caused by soil-borne tomato early blight (Gómez-Rodrıguez et al., 2003), and the intercropping of garlic and cruciferous vegetables reduced the occurrence of soil-borne white rot of garlic (Tamire and Chemeda, 2007). Combatting the degree of plant cell peroxidation is an important mechanism to control crop diseases. Plants themselves primarily achieve this by improving the activity of antioxidant enzymes and enhancing the ability of cells to resist penetration (Zulfiqar et al., 2020). Numerous studies have shown that intercropping can improve the antioxidant capacity and cellular stability of crops to combat the damage from peroxidation and control the occurrence of disease. For example, rice and watermelon intercropping can control the occurrence of watermelon blight by enhancing the activity of watermelon root defense enzymes and the expression of defense genes and reducing the content of H_2_O_2_ and O_2_^−^ in watermelon roots (Ren et al., 2016). Liu et al. (2019) found that the level of expression of the SOD gene in cucumber significantly increased after wheat/cucumber, which enhanced the resistance of cucumber to disease. Not only that, but intercropping can also control the occurrence of disease by improving the root secretion composition of the host crop and reducing the content of autotoxins in it. For example, rice and watermelon intercropping can reduce the composition of the chemical substance secreted by the root system of watermelon phthalic acid and ferulic acid contents and thus control the occurrence of watermelon blight (Lv et al., 2018). The focus of these studies was primarily on the impact of intercropping on the resistance of cash crops, and there are few studies on food crops. Legume crops, in particular, are rarely reported.

Faba bean is an important winter legume crop that is rich in protein and energy and is the main raw material for feed and food. It is widely cultivated worldwide (Siddiqui et al., 2015). However, the long-term continuous planting of faba beans can cause a high incidence of faba bean wilt, which will seriously affect the quality of faba beans (Guo et al., 2020). In Southwest China, the planting pattern of faba bean–wheat intercropping is often used to control the occurrence of FOF. Most of the research on the mechanism of faba bean–wheat intercropping to control the occurrence of faba bean wilt has been conducted in pot experiments, and such studies are rarely conducted in actual farmland. In the early pot experiments, the research group found that the intercropping of faba beans and wheat can effectively improve the resistance of faba bean tissues, increase the diversity of microorganisms in the rhizosphere soil, and alleviate the autotoxicity of faba beans to control the occurrence of faba bean *Fusarium* wilt (Dong et al., 2013). However, this disease has not been explored from the perspective of physiological and biochemical resistance and the expression of genes coding for antioxidant enzymes. Earlier research found that the content of benzoic acid and cinnamic acid in the rhizosphere soil of monocropping faba bean was significantly higher than that of other phenolic acids (Guo et al., 2020). Therefore, we hypothesized that benzoic acid and cinnamic acid are important autotoxic substances that promote the occurrence of faba bean *Fusarium* wilt. Through field and pot experiments, the mechanism by which benzoic acid and cinnamic acid increase the risk of faba bean *Fusarium* wilt, and the potential mechanism of wheat and faba bean intercropping to control faba bean *Fusarium* wilt were explored from the perspective of biochemical resistance and the expression of antioxidant genes.

## Materials and methods

### Experimental materials

The faba bean (*Vicia faba* L.) variety 89-147 and Yunmai 53 wheat (*Triticum aestivum* L.) in this study were purchased from the Yunnan Academy of Agricultural Sciences, Kunming, China.

Analytical benzoic acid and cinnamic acid were purchased from China National Pharmaceutical Group Shanghai Medical Devices Co., Ltd. (Shanghai, China).

FOF was isolated from monoculture faba bean fields by the Plant Microbiology Laboratory of Yunnan Agricultural University. Soil measuring 25 g was taken, 225 ml of water was diluted 10 times, and then 1 ml of diluent was absorbed and placed on a plate. About 15 ml of sterilized pentachloronitrobenzene (PCNB) medium, which was cooled to about 45°C, was poured into the plate, mixed horizontally, and incubated at 25°C–28°C for 7 days. The conidia and colony morphology of FOF are shown in **Figure S1**.

The fungus was transferred to potato dextrose agar (PDA) medium and cultured at 28°C for 7 days. The colonies of *F. oxysporum* cultured on PDA for 7 days were washed with sterile water, filtered through four layers of gauze to collect the spores, and diluted into a spore suspension of 1 × 10^6^ CFU/ml.

#### Field test

The field test was conducted in Efeng Village, Eshan County, Yuxi City, southern Yunnan Province (24°11′N, 102°24′E, 1,540 m above sea level) from October 2019 to May 2020 and October 2020 to May 2021. The local climate is a mid-subtropical semi-humid cold winter. The plateau monsoon climate zone has a mild climate and ample sunshine. The previous crop in the test field was leeks (*Allium ampeloprasum*); the soil type was paddy soil, and the 0–20-cm plow layer soil contained organic matter 28.6 g·kg^−1^, total nitrogen 2.6 g·kg^−1^, total phosphorus 0.75 g·kg^−1^, total potassium 18.2 g·kg^−1^, alkaline hydrolysis nitrogen 108 mg·kg^−1^, available phosphorus 34.2 mg·kg^−1^, and available potassium 97.4 mg·kg^−1^ at pH 6.7.

The experiment was a single-factor design, with two planting modes: monocropping of faba bean and intercropping of faba bean and wheat. The area of each plot is 32.4 m^2^. Each plot has three replicates. A total of six plots were designed. Faba beans are sown in holes, with a spacing of 0.15 m for each plant and 0.3 m for each row. The sowing method of wheat is sowing, with a spacing of 0.24 m for each plant and 0.2 m for each row. The spacing between rows of faba bean wheat is 0.3 m. A total of 24 wheat plants were planted in each row. A total of 18 rows of beans were planted in the faba bean monoculture plot. The row ratio of faba bean to wheat was 2:6 in the intercropping plot. A total of eight rows of faba bean and 18 rows of wheat were planted.

The rate of application of the nitrogen fertilizer for the single and intercropping faba bean plots was 90 kg·hm^−2^ (urea). Phosphate fertilizer was applied at a rate of 100 kg·hm^−2^ (P_2_O_5_), and the rate of application of potassium fertilizer was 100 kg·hm^−2^ (K_2_O). Fertilizer was applied as a base fertilizer at the time of planting.

### Investigation method of faba bean *Fusarium* wilt

#### Field experiment

Three surveys were conducted during the branching, flowering, and maturity stages of faba beans. The specific selection of faba beans was to randomly select five points along the diagonal in the faba bean monoculture plot, with two plants per point, for a total of 10 plants. In the intercropping plot, three points were selected in the first planting zone, and two points were selected in the second planting zone. The specific locations of selected points are shown in **Figure S4**. Two plants were studied at each point, for a total of 10 plants.

After the survey, the incidence and disease index of the *Fusarium* wilt of faba bean were calculated. The degree of disease was divided into five levels (**Figure S2**): 0 means no infection. Level 1 means the initial symptoms of wilt; the base of the stem or root (except the main root) has mild plaque or discoloration. Level 2 means the disease is at the base or root of the stem, but not adjacent to it. Level 3 means 1/3–1/2 of the stem base or roots appear diseased, discolored, or wilted, and the lateral roots are significantly reduced. Level 4 means the stem base is surrounded by lesions, or most of the roots are discolored and wilted. Level 5 means the plant has completely withered and died. The incidence and disease index of *Fusarium* wilt were calculated according to the following formulas.


$$\text{Incidence }=\frac{\text{Number of diseased plants}}{\text{total number of plants investigated}}\times 100\%$$

$$\text{Disease index}=\frac{\text{Σ}\left(\text{Number of diseased plants at each level}\times \text{ level}\right)}{\text{The highest level }\times \text{total number of plants investigated}}\times 100\%$$

#### Rhizosphere soil collection and determination of phenolic acid contents

Soil samples from the faba bean rhizosphere were collected 60 days after the faba bean was sown. After collecting 15 faba bean plants in each plot, the rhizosphere soil was mixed and stored in an ice box for further analysis. A total of 25 g of soil was removed from the ice box and placed in an Erlenmeyer flask, and 25 ml of a solution of 1 mol·L^−1^ of NaOH was added and incubated for 24 h. The sample was then centrifuged at 8,000 rpm for 10 min. The pH of the supernatant was adjusted to 2.5 with 12 mol·L^−1^ of HCl and then incubated for 2 h. After incubation, the sample was centrifuged again at 8,000 rpm for 10 min, and the supernatant of the second centrifugation was stored at 4°C for future use. After the sample was filtered through a 0.45-µm membrane, it was analyzed by high-performance liquid chromatography (HPLC) (Agilent 1260 Infinity, Agilent Technologies, Santa Clara, CA, USA). Chromatographically pure *p*-hydroxybenzoic acid, cinnamic acid, syringic acid, ferulic acid, benzoic acid, and salicylic acid were used as standards. The conditions of HPLC included a Kinetex column (Phenomenex, Torrance, CA, USA), 2.6 μm, 4.6 × 100 mm; a column temperature of 30°C; an injection volume of 10 μl; a 280-nm diode array detector (DAD); a flow rate of 0.5 ml·min^−1^; and a mobile phase of A = methanol (chromatographic grade) and B = 0.1% aqueous phosphoric acid. The separation conditions were as follows: mobile phase B 80% (0 min) → 5% (15.0 min) → 5% (18.0 min) → 80% (18.5 min) → 0% (20.0 min) → stop (25.0 min) for a gradient elution. The type of phenolic acid was determined based on its retention time, and the content of each phenolic acid was calculated using an external standard.

#### Pot experiment

A hydroponic experiment using faba beans and wheat was conducted in the greenhouse of Yunnan Agricultural University from October 2020 to May 2021 under monocropping (M) (six faba beans per pot) or intercropping (I) (three faba beans and nine wheat plants per pot) modes under no stress (CK), pathogen alone stress (F), pathogen and 50 mg·L^−1^ of benzoic acid dual stress (F+50B), pathogen and 50 mg·L^−1^ of cinnamic acid dual stress (F+50C), double stress of pathogen and 100 mg·L^−1^ of benzoic acid (F+100B), double stress of pathogen and 100 mg·L^−1^ of cinnamic acid (F+100C), dual stress of pathogen and 200 mg·L^−1^ of benzoic acid (F +200B), pathogen and 200 mg·L^−1^ of cinnamic acid double stress faba bean (F+200C) treatment. The faba bean and wheat were grown for 14 h:10 h (light:dark) at 22°C–26°C each day. Each treatment was conducted in triplicate.

Faba bean seeds were soaked at room temperature for 24 h and germinated in sterile quartz sand at 25°C. The seedlings were transplanted into 3-L plastic pots (upper diameter 25 cm, lower diameter 13 cm, and 16 cm high) after they had grown four to six leaves and were approximately 10 cm high. The pots contained different concentrations of benzoic acid or cinnamic acid and 2 L of 1 × 10^6^ ml^−1^ of Hoagland nutrient solution of a FOF spore suspension. The same concentration of benzoic acid or cinnamic acid and 1 × 10^6^ ml^−1^ of FOF spore suspension was replaced every 3 days in Hoagland nutrient solution. The position of the pots was randomly arranged. There were replicates, giving a total of 48 pots in a randomized block design. The nutrient solution was stored in the pot and continuously ventilated with a pump.

### Determination of biochemical indices of faba bean plants

In the field test, samples were taken 60 days (fruiting period) after the faba beans were sown, and the samples were taken after the faba beans had grown in the pot experiment for 45 days (fruiting period). Three fresh root samples were collected from each treatment; SOD and CAT activities, and contents of H_2_O_2_ and O_2_^−^ were measured. All the experiments utilized kits from Sino Best Biological Technology Co., Ltd. (Shanghai Best Biological Technology Co., Ltd., Shanghai, China) following the manufacturer’s instructions: O_2_^−^ (Art. No. YX-C-A407, specification 50T/48S), H_2_O_2_ (Art. No. YX-C-A400, specification 50T/48S), SOD (Art. No. YX-C-A500, specification 50T/48S), and CAT (Art. No. YX-C-A501, specification 50T/48S).

### Determination of the level of expression of genes coding for antioxidative enzymes in the faba bean root system

In the field experiment, samples were taken 60 days (fruiting period) after the faba beans were sown, and the samples were collected after 45 days (fruiting period) in the pot experiment. The roots of faba beans were quickly placed in liquid nitrogen and stored for each repetition of three faba beans. Fluorescence quantitative PCR was used to detect the expression of Vf SOD and Vf CAT in the faba bean roots. Vf reference genes were cyclophilin and eukaryotic elongation factor 1-alpha (Gutierrez et al., 2011). The specific primer sequences and hypothetical functions of the genes tested are listed in **Tables S1–S5** in the **Supplementary Material**.

#### Sample preparation and transmission electron microscopy

Samples were taken 1–2 cm away from the root tip with a length of approximately 5 mm. The samples were fixed with glutaraldehyde, placed at room temperature for 2 h, transferred to 4°C, and rinsed three times with phosphate-buffered saline (PBS) (0.1 mol·L^−1^, pH 7.4) for 15 min each. The samples were fixed at room temperature in 1% osmium in 0.1 mol·L^−1^ of phosphate buffer (pH 7.4) for 5 h and then dehydrated. 812 resin was used for embedding, and sections were prepared using a microtome (Leica UC7, Wetzlar, Germany), double stained with uranium and lead, dried overnight at room temperature, and observed by transmission electron microscopy (TEM) (HT7700; Hitachi, Tokyo, Japan). Three replicates were selected for each treatment, and three slides were made and observed three times.

### Statistical analysis

Data on *Fusarium* wilt incidence, disease index, H_2_O_2_ O_2_^−^ content, enzyme, and gene expression are the results of the mean ± standard error of three biological replicates. The data were analyzed using SPSS v. 20.0 (IBM, Inc., Armonk, NY, USA). All the figures were plotted using Origin 2018 (OriginLab, Northampton, MA, USA). Significant differences between treatments were evaluated using a single-factor analysis of variance (ANOVA) followed by a Tukey’s test at the 5% probability level.

For ultrastructure, three replicates were selected for each treatment, and three slides were made and observed three times. However, due to too many treatments in this manuscript, we chose a representative picture.

## Results

### The field experiment on the control of faba bean *Fusarium* wilt by intercropping faba bean and wheat

**Figure 1:**
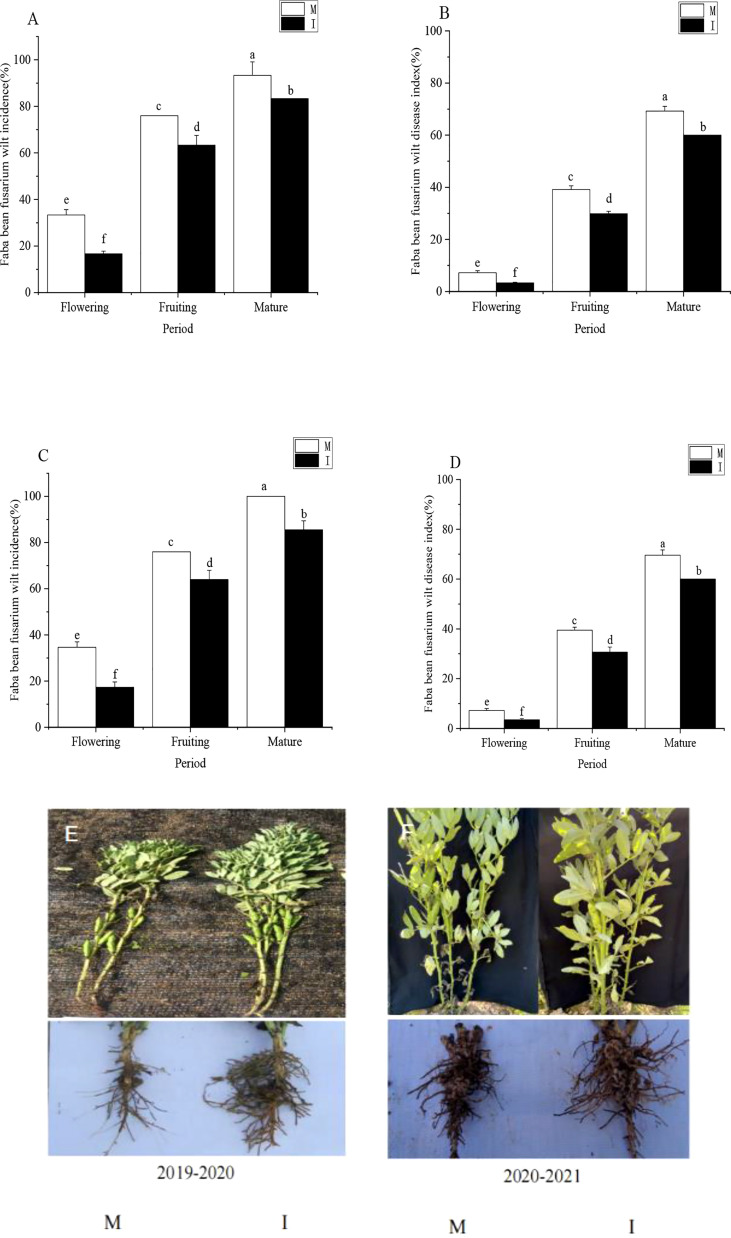
**(A)** Incidence and **(B)** disease index of *Fusarium* wilt in faba beans at different stages of growth from October 2019 to May 2020. **(C)** Incidence and **(D)** disease index of *Fusarium* wilt in faba beans at different stages of growth from October 2020 to May 2021. Phenotypic symptomatic pictures of monocropping **(E)** and intercropping **(F)** faba beans in the 2019–2020 field experiment and 2020–2021 field experiment. Data represent mean ± standard error of three biological replicates. M, faba bean monocropping; I, wheat–faba bean intercropping. The data represent the mean ± standard error of three biological replicates. Different letters for each index indicate significant differences at *p* < 0.05 (Tukey’s test).

In the 2019–2020 field experiment and 2020–2021 field experiment, the incidence of *Fusarium* wilt at the maturity stage was significantly higher than that at the fruiting period (**Figures 1A, C**). The incidence of *Fusarium* wilt in the fruiting period was significantly higher than that at the flowering stage. The disease index of *Fusarium* wilt during the fruiting period was significantly higher than that at the flowering stage, and the disease index of *Fusarium* wilt at the maturity stage was significantly higher than that during the fruiting period (**Figures 1B, D**). The incidence and disease index of faba bean *Fusarium* wilt increased gradually with time.

In the 2019–2020 field experiment, intercropping faba beans and wheat effectively reduced the *Fusarium* wilt of faba beans during the flowering period and significantly reduced the incidence and disease index of *Fusarium* wilt by 50% and 53%, respectively. Intercropping faba beans and wheat effectively reduced the *Fusarium* wilt of faba beans during the fruiting period and significantly reduced the incidence and disease index of *Fusarium* wilt compared with monocropping faba beans by 17% and 25%, respectively. Intercropping faba beans and wheat effectively reduced the *Fusarium* wilt of faba beans during the maturity period and significantly reduced the incidence and disease index of *Fusarium* wilt compared with monocropping faba beans at 11% and 15%, respectively (**Figures 1A, B**).

In the 2020–2021 field experiment, intercropping faba beans and wheat effectively reduced the *Fusarium* wilt of faba beans during the flowering period and significantly reduced the incidence and disease index of *Fusarium* wilt by 50% and 52%, respectively. Intercropping faba beans and wheat effectively reduced the *Fusarium* wilt of faba beans during the fruiting period and significantly reduced the incidence and disease index of *Fusarium* wilt compared with monocropping faba beans by 16% and 25%, respectively. Intercropping faba beans and wheat effectively reduced the *Fusarium* wilt of faba beans during the maturity period and significantly reduced the incidence and disease index of *Fusarium* wilt compared with monocropping faba beans 14% and 15%, respectively (**Figures 1C, D**).

Phenotypic symptomatic pictures of monocropping and intercropping faba beans in the 2019–2020 field experiment and 2020–2021 field experiment are shown in **Figures 1E, F**.

### H_2_O_2_ and O_2_^−^ contents in faba bean roots under monocropping and intercropping field experiments

**Figure 2:**
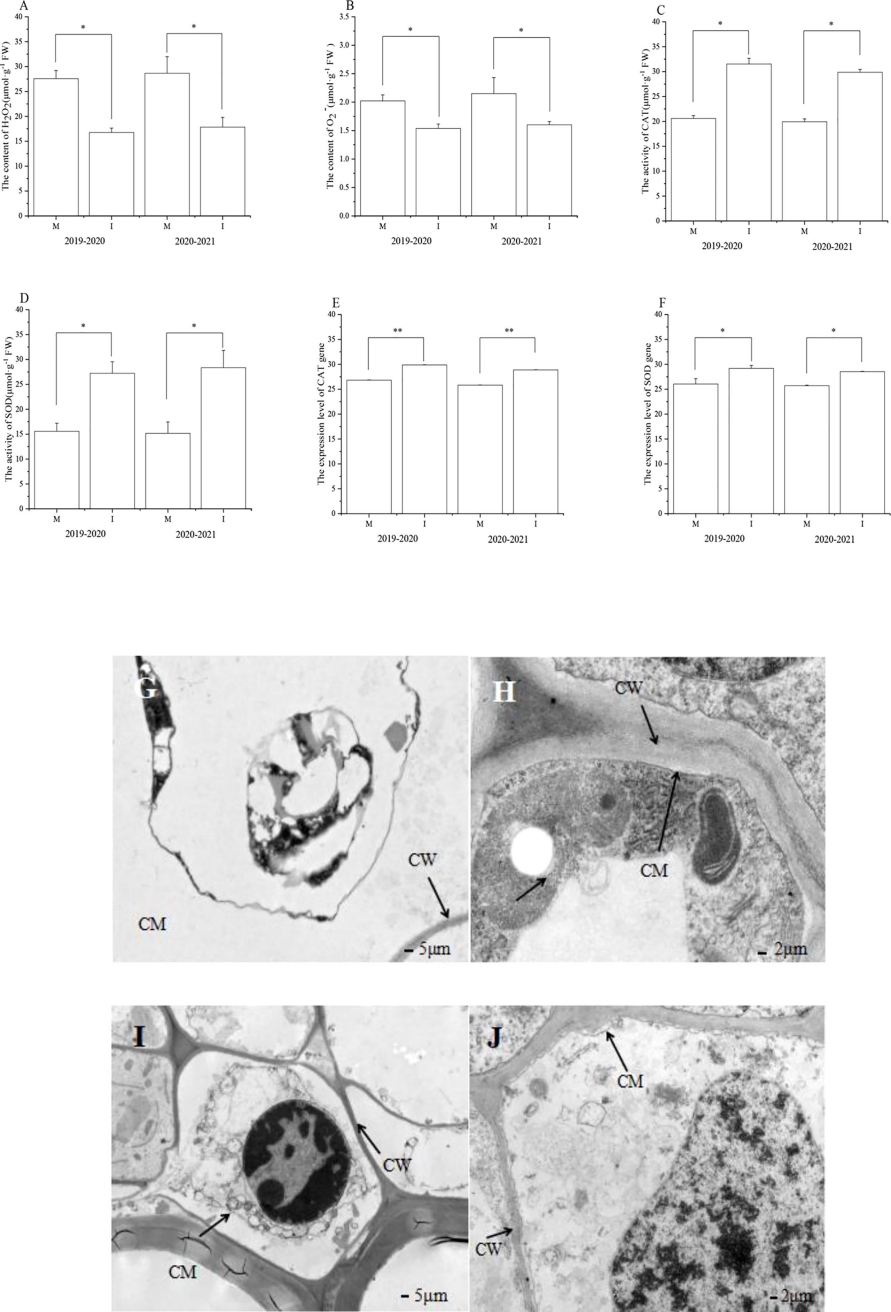
In the field experiment, **(A)** H_2_O_2_ and **(B)** O_2_^−^contents, **(C)** CAT and **(D)** SOD activity, **(E)** CAT and **(F)** SOD level of expression, and **(G–J)** ultrastructure of faba bean (*Vicia faba*) root system under different planting modes was studied. M, faba bean monocropping; I, wheat–faba bean intercropping; CW, cell wall; CM, cell membrane. The data represent the mean ± standard error of three biological replicates. *p < 0.05 (Tukey’s test). CAT, catalase; SOD, superoxide dismutase.

In the 2019–2020 field experiment, the content of H_2_O_2_ in faba bean roots after wheat intercropping decreased significantly by 39% compared with that of the monocropping faba bean (**Figure 2A**). The content of O_2_^−^ in the roots of faba bean and wheat intercropping was significantly reduced by 24% compared with that of single cropped faba bean (**Figure 2B**).

In the 2020–2021 field experiment, the content of H_2_O_2_ in faba bean roots after wheat intercropping decreased significantly by 38% compared with that of the monocropping faba bean (**Figure 2A**). The content of O_2_^−^ in the roots of faba bean and wheat intercropping was significantly reduced by 26% compared with that of the monocropping faba bean (**Figure 2B**).

### The field experiment on catalase and superoxide dismutase activities of faba bean roots under monocropping and intercropping

In the 2019–2020 field experiment, compared with the monocropping faba bean, the activity of CAT in the root system of faba beans and wheat intercropping increased significantly by 53% (**Figure 2C**). Compared with the monocropping faba bean, the SOD activity in the root system of faba bean and wheat intercropping increased significantly by 75% (**Figure 2D**).

In the 2020–2021 field experiment, compared with the monocropping faba bean, the activity of CAT in the root system of faba beans and wheat intercropping increased significantly by 50% (**Figure 2C**). Compared with the monocropping faba bean, the SOD activity in the root system of faba bean and wheat intercropping increased significantly by 87% (**Figure 2D**).

### In the field experiment, the levels of expression of catalase and superoxide dismutase in the roots of monocropping and intercropping faba bean

In the 2019–2020 field experiment, compared with monocropping faba bean, the expression of CAT in the root system of faba bean and wheat intercropping significantly increased by 11% (**Figure 2E**). In addition, compared with the monocropping faba bean, the level of expression of SOD in the root system of faba bean intercropped with wheat significantly increased by 12% (**Figure 2F**).

In the 2020–2021 field experiment, compared with monocropping faba bean, the expression of CAT in the root system of faba bean and wheat intercropping significantly increased by 12% (**Figure 2E**). In addition, compared with the monocropping faba bean, the level of expression of SOD in the root system of faba bean intercropped with wheat significantly increased by 11% (**Figure 2F**).

### Ultrastructure of monocropping and intercropped faba bean roots in a field experiment

In the 2019–2020 field experiment and 2020–2021 field experiment, cytoplasmic wall separation was obvious in the faba bean roots under monocropping (**Figures 2G, I**). There was almost no cytoplasmic wall separation in the faba bean roots under intercropping (**Figures 2H, J**). The samples were prepared and observed using TEM.

### The field experiment on the content of phenolic acid in root exudates of monocropping and intercropping faba bean plants

**Figure 3:**
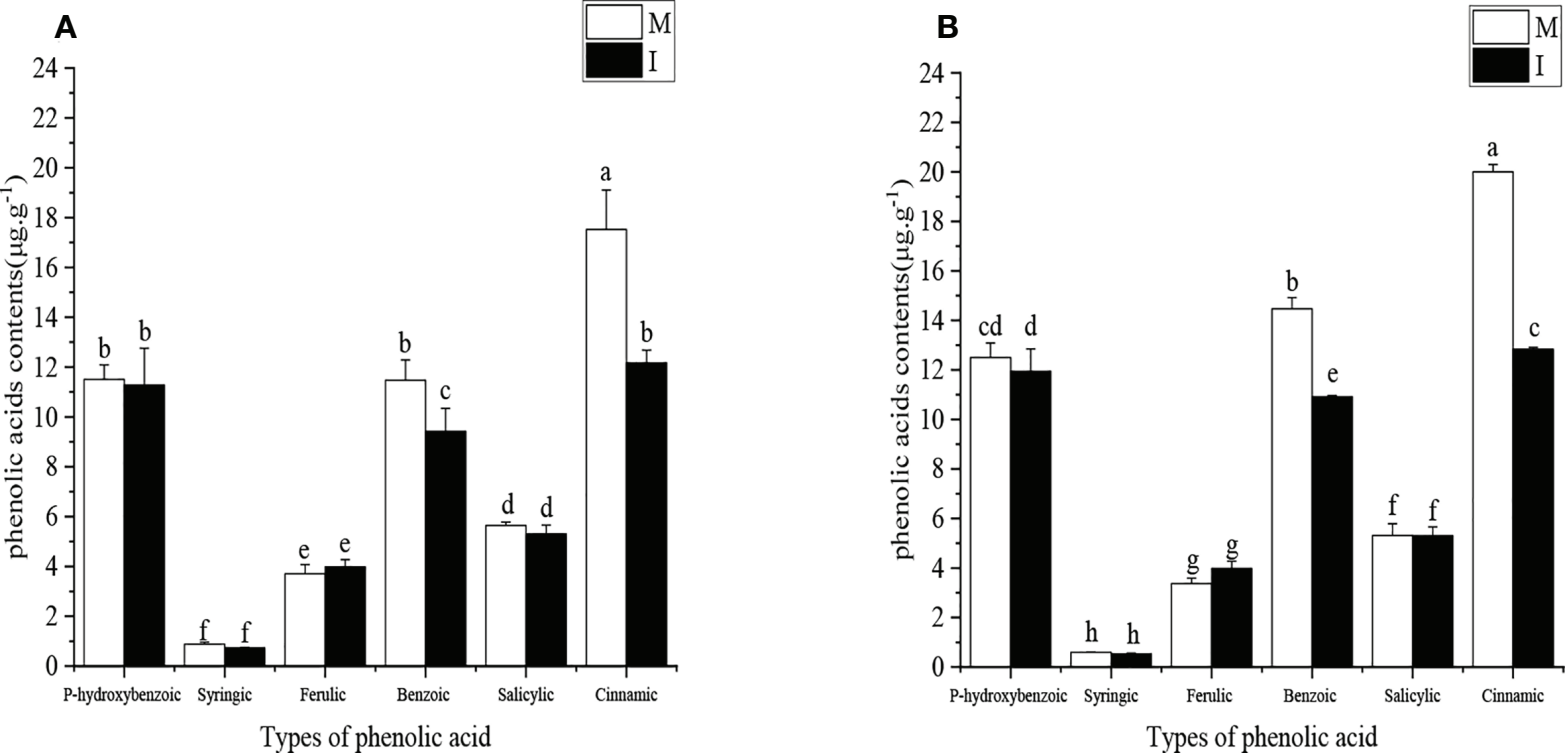
**(A)** The types and contents of phenolic acids in the faba bean (*Vicia faba*) rhizosphere soil under different planting modes in the October 2019 to May 2020 field experiment. **(B)** The types and contents of phenolic acids in the faba bean (*Vicia faba*) rhizosphere soil under different planting modes in October 2020 to May 2021 field experiment. M, faba bean monocropping; I, wheat–faba bean intercropping. The data represent the mean ± standard error of three biological replicates. Different letters for each index indicate significant differences at *p* < 0.05 (Tukey’s test).

The types and contents of phenolic acids in the root exudates of faba bean were determined in the 2019–2020 field experiment and 2020–2021 field experiment (**Figures 3A, B**). Six major phenolic acids were found, namely, *p*-hydroxybenzoic acid, syringic acid, ferulic acid, benzoic acid, salicylic acid, and cinnamic acid. In the 2019–2020 field experiment, compared with faba bean monocropping, the contents of benzoic acid and cinnamic acid in the rhizosphere soil of faba bean decreased by 18% and 31% in wheat and faba bean intercropping, respectively. In the 2020–2021 field experiment, compared with faba bean monocropping, the contents of benzoic acid and cinnamic acid in the rhizosphere soil of faba bean decreased by 25% and 36% in wheat and faba bean intercropping, respectively. The contents of benzoic acid and cinnamic acid in the monocropping mode were significantly higher than those of the other phenolic acids.

### Effects of different concentrations of exogenous benzoic acid and cinnamic acid on the incidence and disease index of *Fusarium* wilt of faba bean inoculated with *Fusarium oxysporum* and the alleviating effect of intercropping

Under the monoculture cultivation mode and compared with F, F+50B, F+100B, and F+200B significantly increased the incidence of *Fusarium* wilt by 57%, 83%, and 144%, respectively (**Table 1**). Compared with F, F+50B, F+100B, and F+200B significantly increased the *Fusarium* wilt disease index by 95%, 115%, and 161%, respectively (**Table 1**).

**Table 1:** Effects of different concentrations of benzoic acid on incidence and disease index of faba bean (*Vicia faba*) *Fusarium* wilt under different cropping modes.

	CK		F		F+50B		F+100B		F+200B	
	M	I	M	I	M	I	M	I	M	I
Incidence of *Fusarium* wilt (%)	0.00g	0.00g	33.33e ± 0	19.45f ± 0.69	52.26c ± 2.86	45.55d ± 3.85	61.11b ± 4.81	50cd ± 0	81.48a ± 6.41	63.11b ± 6.16
*Fusarium* wilt index (%)	0.00g	0.00g	27.17e ± 2.46	18.44f ± 1.35	53.08c ± 1.3	25e ± 1.67	58.28b ± 2.58	43.75d ± 3.25	70.83a ± 2.5	55.56bc ± 4.45

The data represent the mean ± standard error of three biological replicates. Different letters for each index indicate significant differences at p < 0.05 (Tukey’s test).

F, *Fusarium oxysporum* f. sp. *fabae*.

Compared with monoculture, intercropping with F, F+50B, F+100B, and F+200B significantly reduced the incidence of *Fusarium* wilt by 42%, 13%, 19%, and 23%, respectively (**Table 1**). Under the F, F+50B, F+100B, and F+200B treatments, intercropping significantly reduced the disease index of *Fusarium* wilt by 32%, 53%, 25%, and 22%, respectively (**Table 1**).

Under the monoculture cultivation mode compared with F, F+50C, F+100C, and F+200C significantly increased the incidence of *Fusarium* wilt by 44%, 111%, and 178%, respectively (**Table 2**). In the monocropping mode, compared with F, F+50C, F+100C, and F+200C significantly increased the disease index of *Fusarium* wilt by 28%, 91%, and 159%, respectively (**Table 2**).

**Table 2:** Effects of different concentrations of cinnamic acid on incidence and disease index of faba bean (*Vicia faba*) *Fusarium* wilt under different cropping modes.

	CK		F		F+50C		F+100C		F+200C	
	M	I	M	I	M	I	M	I	M	I
Incidence of *Fusarium* wilt (%)	0.00f	0.00f	33.33d ± 0	19.45e ± 0.69	48.15c ± 3.41	33.33d ± 0	70.37b ± 2.86	51.85c ± 3.21	92.59a ± 6.41	74.07b ± 4.81
*Fusarium* wilt index (%)	0.00f	0.00f	27.17d ± 2.46	18.44e ± 1.35	34.81c ± 2.57	25.19d ± 1.28	51.85b ± 5.59	37.03c ± 1.28	70.37a ± 3.39	54.07b ± 4.41

The data represent the mean ± standard error of three biological replicates. Different letters for each index indicate significant differences at p < 0.05 (Tukey’s test).

F, *Fusarium oxysporum* f. sp. *fabae*.

Compared with monocropping, intercropping with F, F+50C, F+100C, and F+200C significantly reduced the incidence of *Fusarium* wilt by 42%, 31%, 26%, and 20%, respectively (**Table 2**). Compared with monocropping, F, F+50C, F+100C, and F+200C intercropping significantly reduced the disease index of *Fusarium* wilt by 32%, 28%, 29%, and 23%, respectively (**Table 2**).

Phenotypic symptomatic pictures of faba bean (*V. faba*) with different concentrations of exogenous benzoic acid and cinnamic acid under different cropping modes are shown in **Figure S3**.

### Effects of exogenous benzoic acid and cinnamic acid at different concentrations on reactive oxygen species in the roots of faba bean inoculated with *Fusarium oxysporum* and its alleviation by intercropping

**Figure 4:**
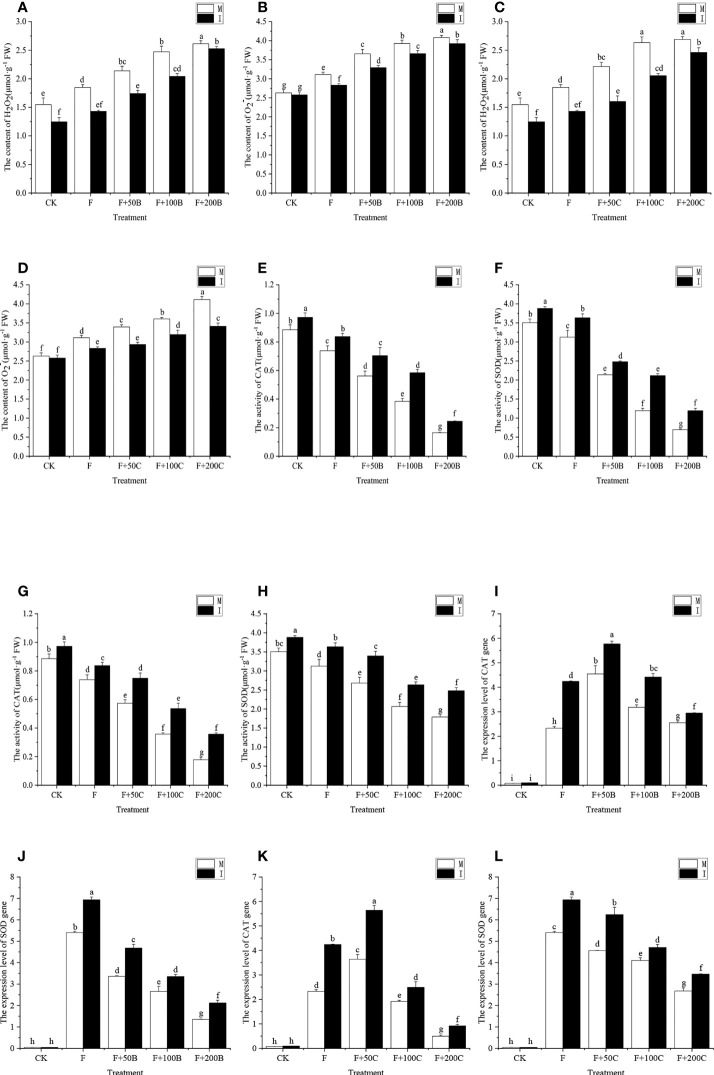
Effects of different concentrations of benzoic acid on root **(A)** H_2_O_2_ and **(B)** O_2_^−^ of faba bean (*Vicia faba*) inoculated with F under different cropping modes. Effects of different concentrations of cinnamic acid on root **(C)** H_2_O_2_ and **(D)** O_2_^−^ of root faba bean inoculated with F under different cropping modes. Effects of different concentrations of benzoic acid on SOD activity in the root modes **(E)** and CAT **(F)** of faba bean (*Vicia faba*) inoculated with F under different cropping modes. Effects of different concentrations of cinnamic acid on SOD **(G)** and CAT **(H)** in root modes of faba bean inoculated with F under different cropping systems. Effects of different concentrations of benzoic acid on **(I)** CAT and **(J)** SOD expression in the root modes of faba bean (*Vicia faba*) inoculated with F under different cropping modes. Effects of different concentrations of cinnamic acid on the levels of expression of **(K)** CAT and **(L)** SOD in the root system of faba bean inoculated with FOF under different cropping modes. Different letters for each index indicate significant differences at *p* < 0.05 (Tukey’s test). F, *Fusarium oxysporum* f. sp. *fabae*; H_2_O_2_, hydrogen peroxide; O_2_^−^, superoxide anion; CAT, catalase; SOD, superoxide dismutase; M, faba bean monocropping; I, wheat–faba bean intercropping. The data represent the mean ± standard error of three biological replicates.

Inoculation with FOF and application of exogenous benzoic acid can significantly increase the contents of H_2_O_2_ and O_2_^−^ in faba bean roots, with a concentration effect (**Figures 4A, B**). In monoculture, compared with CK, the contents of H_2_O_2_ under the F, F+50B, F+100B, and F+200B treatments significantly increased by 19%, 38%, 60%, and 69%, respectively, and the contents of O_2_^−^ significantly increased by 18%, 39%, 49%, and 55%, respectively. In monoculture, compared with F, the activity of H_2_O_2_ increased significantly by 16%, 34%, and 41%, respectively, and the O_2_^−^ content increased significantly by 17%, 26%, and 31% under the F+50B, F+100B, and F+200B treatments, respectively.

Compared with monocropping, faba bean and wheat intercropping reduced the effects of FOF and benzoic acid on the degree of peroxidation of faba bean root. Compared with monocropping, under the CK, intercropping significantly reduced the contents of H_2_O_2_ by 19%; under F, F+50B, F+100B, and F+200B, intercropping significantly reduced the contents of H_2_O_2_ and O_2_^−^ by 23%, 19%, 17%, and 3%, respectively, and O_2_^−^ decreased by 9%, 10%, 7%, and 4%, respectively.

Both inoculation with FOF and application of exogenous cinnamic acid significantly increased the contents of H_2_O_2_ and O_2_^−^ in faba bean roots, with a concentration effect (**Figures 4C, D**). In monoculture, compared with the CK, the content of H_2_O_2_ significantly increased by 19%, 43%, 70%, and 74% under F, F+50C, F+100C, and F+200C, respectively, and the content of O_2_^−^ significantly increased by 18%, 29%, 37% and 57% under F, F+50C, F+100C, and F+200C, respectively. In monoculture, compared with F, the activity of H_2_O_2_ increased significantly by 20%, 42%, and 45% under F+50C, F+100C, and F+200C, respectively, and the content of O_2_^−^ increased significantly by 9%, 16%, and 32%, respectively.

Compared with monocropping, faba bean and wheat intercropping reduced the effects of FOF and cinnamic acid on the degree of peroxidation of faba bean roots. Compared with monocropping, under the CK, intercropping significantly reduced the contents of H_2_O_2_ by 19%; under the F, F+50B, F+100B, and F+200B, intercropping significantly reduced the contents of H_2_O_2_ by 23%, 28%, 22%, and 8%, respectively, while the contents of O_2_^−^ decreased by 9%, 14%, 11%, and 17%, respectively.

### Effects of exogenous benzoic acid and cinnamic acid at different concentrations on the root defense enzymes catalase and superoxide dismutase of faba bean inoculated with *Fusarium oxysporum* and alleviation by intercropping

The inoculation of FOF and exogenous addition of benzoic acid could significantly reduce the activities of CAT and SOD in the root system of faba bean with a concentration effect (**Figures 4E, F**). The F, F+50B, F+100B, and F+200B treatments could significantly reduce the activities of CAT and SOD in the faba bean root system and had a concentration effect. In monoculture, compared with CK, the activity of CAT decreased significantly by 17%, 37%, 57%, and 81% in the F+50B, F+100B, and F+200B treatments, respectively, while the activity of SOD significantly decreased by 11%, 39%, 66%, and 80%, respectively. In monoculture, compared with F, the activity of CAT decreased significantly by 24%, 48%, and 78% in the F+50B, F+100B, and F+200B treatments, respectively, and the activity of SOD decreased significantly by 32%, 62%, and 78%, respectively.

Compared with monocropping, intercropping with faba bean and wheat reduced the effects of FOF and benzoic acid on the activities of antioxidative enzymes in faba bean roots. Intercropping with wheat and faba bean significantly increased the activities of CAT by 10%, 13%, 25%, 52%, and 49% and those of SOD by 11%, 16%, 16%, 77%, and 71% for CK, F, F+50B, F+100B, and F+200B, respectively.

The exogenous inoculation of FOF and addition of cinnamic acid could significantly reduce the activities of CAT and SOD in the root system of faba bean with a concentration effect (**Figures 4G, H**). In monoculture, compared with CK, the activity of CAT decreased significantly by 17%, 35%, 60%, and 80% under the F, F+50C, F+100C, and F+200C treatments, respectively, and the activity of SOD decreased significantly by 11%, 24%, 41%, and 49% under the F, F+50C, F+100C, and F+200C treatments, respectively. In monoculture, compared with F, the activity of CAT decreased significantly by 24%, 48%, and 78% under the F, F+50C, F+100C, and F+200C treatments, respectively, and the activity of SOD decreased significantly by 22%, 52%, and 76% under F, F+50C, F+100C, and F+200C, respectively.

Compared with monocropping, intercropping with faba bean and wheat reduced the effects of FOF and cinnamic acid on the activities of defensive enzymes in faba bean roots. Intercropping with wheat and faba bean significantly increased the activities of CAT by 10%, 13%, 31%, 50%, and 101% and those of SOD by 11%, 16%, 27%, 28%, and 39% for CK, F, F+50C, F+100C, and F+200C, respectively.

### Effects of different concentrations of exogenous benzoic acid and cinnamic acid on the level of expression of catalase and superoxide dismutase of faba bean plants inoculated with *Fusarium oxysporum* and the alleviation by intercropping

In monoculture, compared with the CK, the F, F+50B, F+100B, and F+200B treatments significantly increased the levels of expression of CAT and SOD in plants (**Figures 4I, J**). In monoculture, compared with F, the expression of CAT increased in the F+50B, F+100B, and F+200B treatments, significantly increasing by 95%, 37%, and 10%, respectively. In monoculture, compared with F, the level of expression of SOD in plants treated with F+50B, F+100B, and F+200B decreased significantly by 38%, 51%, and 75%, respectively.

Compared with monocropping, the levels of expression of CAT and SOD in the root system of faba bean and wheat intercropping in the F, F+50B, F+100B, and F+200B treatments increased significantly; the levels of expression of CAT increased by 82%, 27%, 39%, 15%, respectively; the levels of expression of SOD increased by 28%, 39%, 26%, 57% respectively.

In monoculture, compared with the CK, the F, F+50C, F+100C, and F+200C treatments significantly increased the levels of expression of CAT and SOD (**Figures 4K, L**). In monoculture, compared with F, the level of expression of CAT in the F+50C treatment significantly increased by 56%. In monoculture, in the F+100C and F+200C treatments, compared with the F treatment, the levels of expression of CAT significantly decreased by 18% and 79%, respectively. In monoculture, compared with F, the level of expression of SOD in the F+50C, F+100C, and F+200C treatments significantly increased by 16%, 24%, and 50%, respectively.

Compared with monocropping, the levels of expression of CAT and SOD in the root system of faba bean and wheat intercropping in the F, F+50C, F+100C, and F+200C treatments increased significantly, and the levels of expression of CAT increased by 82%, 55%, 30%, and 85%, respectively. SOD expression increased by 28%, 37%, 15%, and 29% respectively.

### Effects of exogenous benzoic acid and cinnamic acid at different concentrations on the ultrastructure of faba bean plants inoculated with *Fusarium oxysporum* and alleviation by intercropping

**Figure 5:**
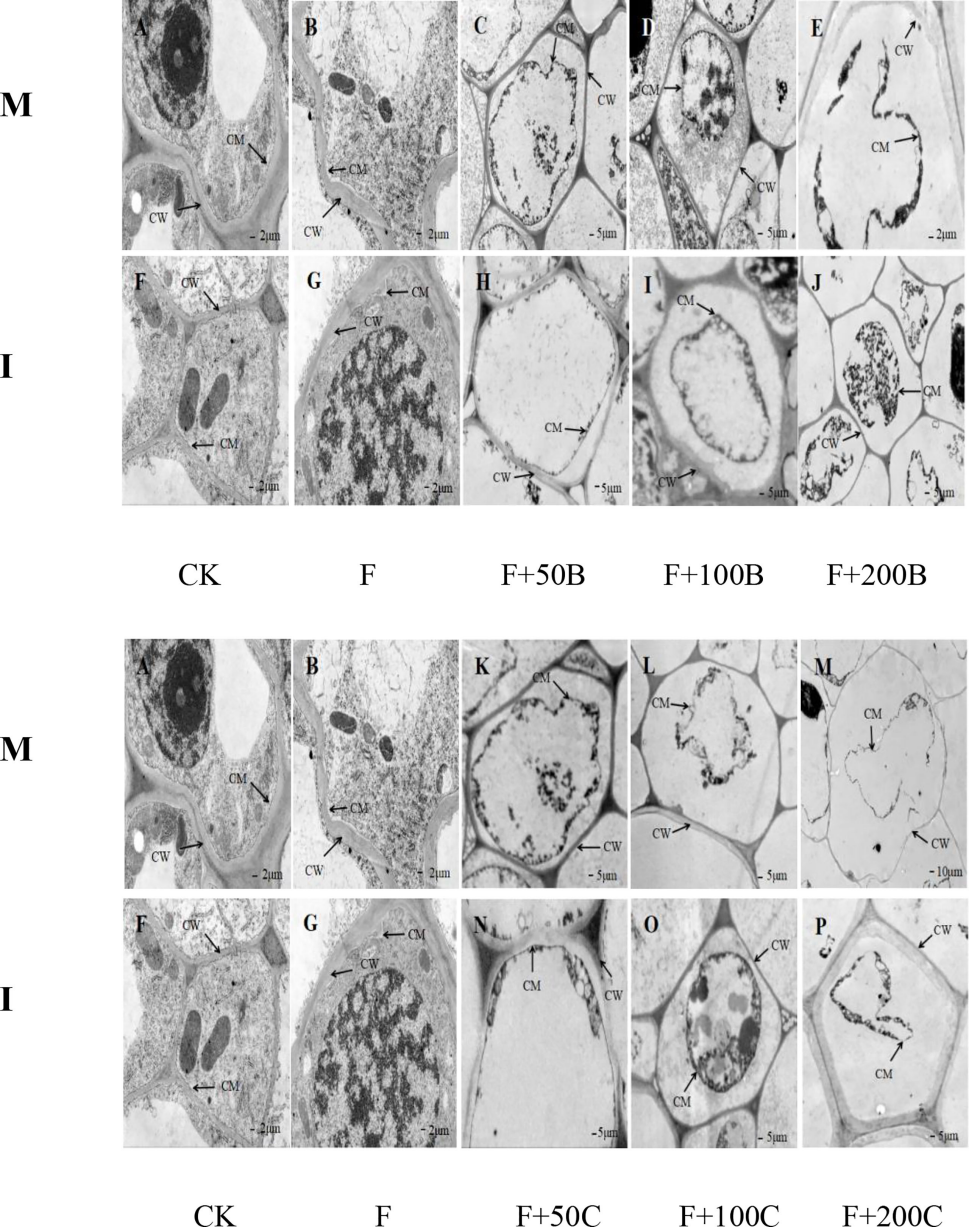
Effects of different concentrations of benzoic acid on ultrastructure in a root of faba bean (*Vicia faba*) inoculated with F under different cropping modes. Effects of different concentrations of cinnamic acid on ultrastructure in a root of faba bean (*Vicia faba*) inoculated with F under different cropping modes. M, faba bean monocropping; I, wheat–faba bean intercropping; CW, cell wall; CM, cell membrane; F, *Fusarium oxysporum* f. sp. fabae. **(A)** Monocropping faba bean under CK treatment. **(B)** Monocropping faba bean under inoculation FOF. **(C)** Monocropping faba bean under inoculated FOF and exogenous application of 50 mg·L^−1^ of benzoic acid. **(D)** Monocropping faba bean under inoculated FOF and exogenous application of 100 mg·L^−1^ of benzoic acid. **(E)** Monocropping faba bean under inoculated FOF and exogenous application of 20 mg·L^−1^ of benzoic acid. **(F)** Intercropping faba bean under CK treatment. **(G)** Intercropping faba bean under inoculation FOF. **(H)** Intercropping faba bean under inoculated FOF and exogenous application of 50 mg·L^−1^ of benzoic acid. **(I)** Intercropping faba bean under inoculated FOF and exogenous application of 100 mg·L^−1^ of benzoic acid. **(J)** Intercropping faba bean under inoculated FOF and exogenous application of 200 mg·L^−1^ of benzoic acid. **(K)** Monocropping faba bean under inoculated FOF and exogenous application of 50 mg·L^−1^ of cinnamic acid. **(L)** Monocropping faba bean under inoculated FOF and exogenous application of 100 mg·L^−1^ of cinnamic acid. **(M)** Monocropping faba bean under inoculated FOF and exogenous application of 200 mg·L^−1^ of cinnamic acid. **(N)** Intercropping f faba bean under inoculated FOF and exogenous application of 50 mg·L^−1^ of cinnamic acid. **(O)** Intercropping faba bean under inoculated FOF and exogenous application of 100 mg·L^−1^ of cinnamic acid. **(P)** Intercropping faba bean under inoculated FOF and exogenous application of 200 mg·L^−1^ of cinnamic acid.

As shown in **Figure 5**, in the monoculture mode, the root cells of the CK treatment (**Figure 5A**) had an intact cell membrane and no obvious cell membrane separation from the cell wall. The root cells of faba bean treated by F had an intact cell membrane but showed cell membrane slight separation from the cell wall (**Figure 5B**). The root cell membrane of faba bean treated with F+50B had separated from the cell wall (**Figure 5C**). The separation of the cell membrane from the cell wall of faba bean root was further aggravated under the F+100B treatment, and the cell membrane was completely separated from the cell wall (**Figure 5D**). The plasma membrane of faba bean root cells under the F+200B treatment was disrupted; the cytoplasm had drained, and the cells completely died (**Figure 5E**). With the increase of exogenous benzoic acid concentration, the cell membrane separation from the cell wall of faba bean root cells was gradually evident and reached its maximum at a concentration of 200 mg·L^−1^.

No separation of faba bean root cell membrane from cell wall was observed in monoculture and intercropped faba bean root cells under the CK treatment (**Figures 5A, F**). Compared with monoculture, under the F treatment, there was no separation phenomenon of faba bean root cell membrane from the cell wall in intercropping compared with monoculture (**Figure 5G**). Compared with monoculture, under the F+50B treatment, intercropping effectively alleviated the separation of the faba bean root cell membrane from the cell wall (**Figure 5H**). Under the F+100B treatment, the degree of separation of faba bean root cell membrane from cell wall under intercropping was smaller than that under monoculture. Compared with monoculture, the root cells of faba bean under F+200B had an intact cell membrane structure and no plasma membrane disruption. The results showed that intercropping effectively protected the integrity of root cells and alleviated the damage of benzoic acid to the root cells of the faba bean.

The root cell membrane of faba bean treated with F+50C had separated from the cell wall (**Figure 5K**). Under F+100C treatment, the separation of the cell membrane from the cell wall of faba bean root was further aggravated (**Figure 5L**). The plasma membrane of faba bean root cells under the F+200C treatment was disrupted, and cells died completely (**Figure 5M**). With the increase of exogenous cinnamic acid concentration, the cell membrane separation from the cell wall of faba bean root cells was gradually evident and reached its maximum at a concentration of 200 mg·L^−1^.

Compared with monoculture, under the F+50C treatment, intercropping effectively alleviated the separation of the faba bean root cell membrane from the cell wall (**Figure 5N**). Under the F+100C treatment, the degree of separation of faba bean root cell membrane from cell wall under intercropping was smaller than that under monoculture. Intercropping could maintain the whole cell shape of root cells (**Figure 5O**). Compared with monoculture, under the F+200C treatment, intercropping could not maintain the plasma membrane’s complete shape but had a complete cell wall structure and no rupture. The degree of separation of faba bean root cell membrane from cell wall under intercropping was smaller than that under monoculture (**Figure 5P**). The results showed that intercropping effectively protected the integrity of faba bean root cells and alleviated the damage of cinnamic acid to faba bean root cells.

## Discussion

Faba bean *Fusarium* wilt is an important factor restricting faba bean production. Southwest China often adopts faba bean–wheat intercropping to control the occurrence of faba bean wilt. In a 2-year field experiment, we found that faba bean–wheat intercropping significantly reduced the incidence and disease index of *Fusarium* wilt in faba bean. Intercropping significantly reduced the contents of H_2_O_2_ and O_2_^−^ in faba bean plants, increased the activity and level of gene expression of CAT and SOD, and effectively protected the integrity of faba bean root cells. Similar findings have been made in many systems (Hao et al., 2010). This research proved that intercropping can increase crop resistance by regulating the physiological and biochemical processes in crops, thereby reducing the occurrence of diseases.

We also found that compared with single-cropping faba beans, faba bean–wheat intercropping significantly reduced the contents of benzoic acid and cinnamic acid in the rhizosphere of faba bean. Similar findings have been observed in other systems. For example, the tomato–celery–cucumber–cabbage rotation system reduced the content of phenolics in the soil compared to the monocropping cucumber (Zhou et al., 2017). Therefore, we believe that the reduction in phenolic acid content after intercropping decreased the quality of soil carbon resources, which may be related to the changes in soil microbial communities and the reduction of soil-borne pathogens (X Jin et al., 2022). Soil microbial community composition is strongly influenced by tillage practices (Hartman et al., 2018). In addition, it has been shown that the mixed apoplast of multiple plants can alter the abundance and diversity of bacterial and fungal communities in soil microorganisms. Therefore, we think intercropping reduces the abundance of pathogenic bacteria while increasing the abundance of beneficial microorganisms in rhizosphere soil, thus reducing the content of phenolic acids. Ren et al. (2008) found that the contents of four kinds of phenolic acids in the rhizosphere of watermelon were significantly reduced after intercropping, and the incidence of watermelon wilt was significantly reduced. This proved that reducing the content of the phenolic acid autotoxic substance would reduce the incidence of disease. However, the increase in plant resistance will also reduce the occurrence of diseases (Lv et al, 2018). Therefore, we hypothesized that the increase in plant stress resistance was the result of decreasing phenolic acid content in the rhizosphere. Based on this hypothesis, we established a pot experiment to explore the effects of benzoic acid and cinnamic acid on the resistance of faba bean plants.

In 2 years of field experiments, we found that as the continuous cropping years increased, the content of benzoic acid and cinnamic acid increased. In the 2019–2020 field experiment, the content of cinnamic acid in root exudates of monoculture faba bean was 17.526 μg·g^−1^, and the content of benzoic acid in root exudates of monoculture faba bean was 11.47 μg·g^−1^ (**Figure 3A**). In the 2020–2021 field experiment, the content of cinnamic acid in root exudates of monoculture faba bean was 19.999 μg·g^−1^, and the content of benzoic acid in root exudate of monoculture faba bean was 14.47 μg·g^−1^ (**Figure 3B**). This is only the result of 1 year of continuous cropping. In addition, benzoic acid and cinnamic acid are often used as food for soil microorganisms in field experiments, and the consumption of phenolic acids by field microorganisms is huge. The research of Liu et al. (2016) proved this point of view. Therefore, we believe that the actual phenolic acid content produced by plant rhizosphere should be much higher than our measured value. Hence, we designed a gradient of 50, 100, and 200 mg·L^−1^ for the pot experiment. The concentration gradient set in this paper is also consistent with ours (Singh, 2015).

**Figure 6:**
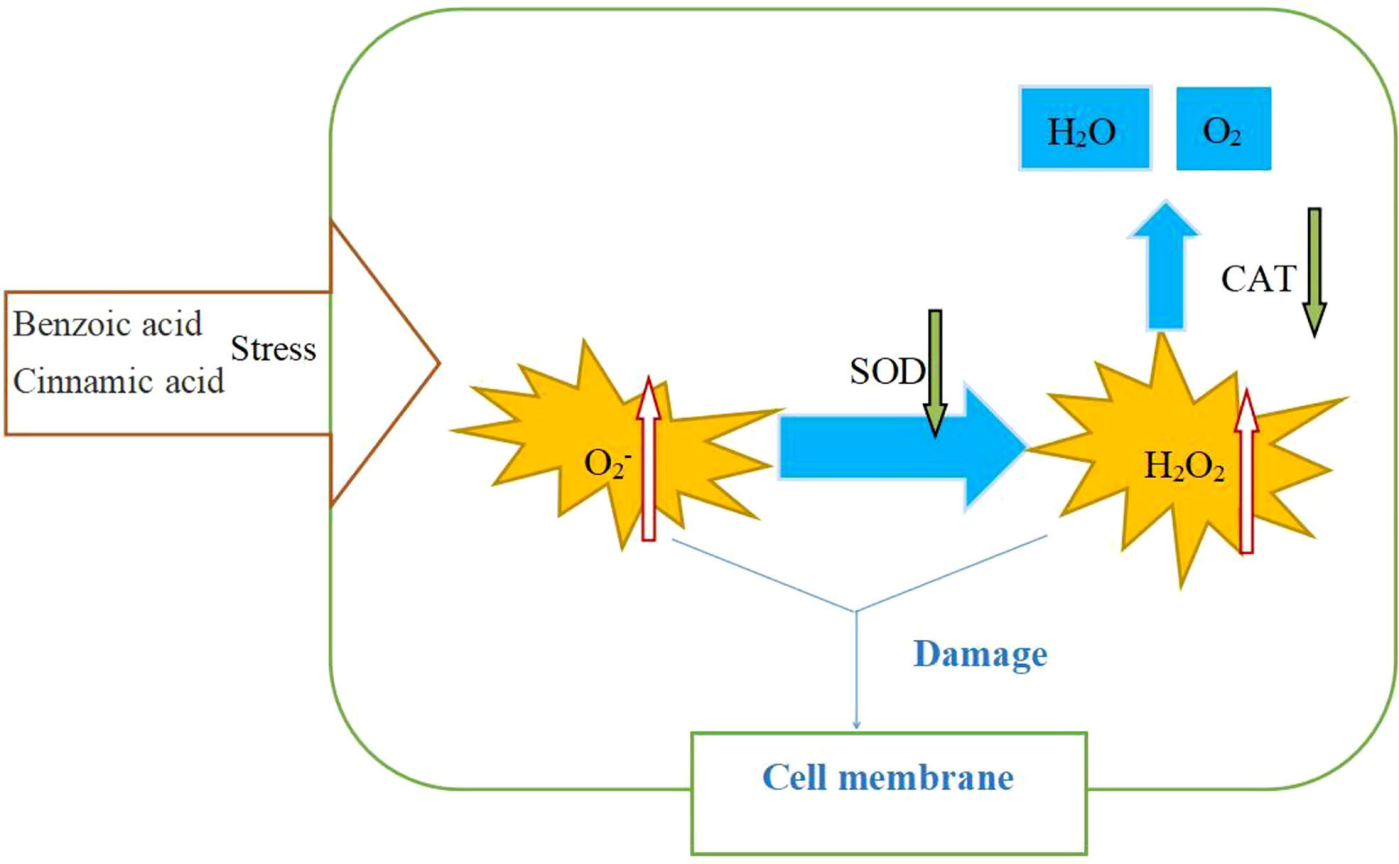
Effects of benzoic acid and cinnamic acid on physiological and biochemical processes of faba bean (*Vicia faba*).

Benzoic acid and cinnamic acid increase the production of reactive oxygen species in plant cells, damaging cell membranes (**Figure 6**), and have been proven to be autotoxic chemicals in many planting systems (Su et al., 2006; Hao et al., 2010). Our results also support this conclusion. We found the incidence and disease index of the *Fusarium* wilt of faba bean increased with the increase in concentrations of benzoic acid and cinnamic acid. Moreover, we found that the exogenous addition of benzoic acid and cinnamic acid induced an outbreak of H_2_O_2_ and O_2_^−^. Excessive H_2_O_2_ and O_2_^−^ can cause oxidative damage to faba bean cells and usually increase the permeability of the cell membrane, destroy the cell wall, and ultimately lead to cell damage and death (Lin et al., 2000). Similar findings were also found in tomatoes treated with exogenous benzoic acid (Singh et al., 2015). This study was conducted by TEM observations that showed that under the monoculture mode, the root cells of faba bean with 50 mg·L^−1^ of benzoic acid and cinnamic acid displayed obvious separation of faba bean root cell membrane from the cell wall. The exogenous addition of 100 mg·L^−1^ of benzoic acid and cinnamic acid further aggravated the separation of the faba bean root cell membrane from the cell wall. The cell membrane had completely separated from the cell wall. After the exogenous addition of 200 mg·L^−1^ of benzoic acid and cinnamic acid, the cell membrane of faba bean root cells was damaged; the cytoplasm had drained, and the cells completely died. These results indicated that benzoic acid and cinnamic acid caused oxidative damage to the cells by increasing the contents of H_2_O_2_ and O_2_^−^ in faba bean plants and destroying the cell membrane structure, thus causing the disease and death of plants.

Generally, plants use the antioxidant enzyme SOD to degrade O_2_^−^, convert O_2_^−^ into H_2_O_2_, and then use CAT to reduce the content of H_2_O_2_ and convert it into H_2_O and O_2_ (Gill and Tuteja, 2010). Zhang et al. (2018) showed that the exogenous addition of autotoxins reduced the level of expression of the SOD gene, resulting in a lack of clearance of ROS and the subsequent death of melon root cells. This is similar to our results. We found that in monoculture mode, F treatment significantly increased the level of expression of the SOD gene and CAT gene compared with the CK. However, high concentrations of benzoic acid and cinnamic acid significantly decreased SOD and CAT gene expression. Therefore, we hypothesized that after inoculation with FOF, the antioxidant system of the faba bean begins to function, and the expression of antioxidant enzymes SOD and CAT increased. The exogenous addition of high concentrations of benzoic acid and cinnamic acid can significantly inhibit the antioxidant capacity of faba beans and destroy the normal physiological metabolic pathways in the tissues of faba beans.

The ability of antioxidant enzymes to scavenge ROS depends not only on the amount of gene expression at the transcription level but also on the amount of protein synthesis at the translational level and post-translational modification regulation (Liang et al., 2011). In our research, we found that the activity of the SOD enzyme in faba beans depends on the level of transcription of SOD. In monoculture mode, compared with the F treatment, different concentrations of exogenous benzoic acid and cinnamic acid could significantly reduce the activity of SOD and had a concentration effect. The results of CAT enzyme activity and gene expression were not consistent. In the monoculture mode, compared with the F treatment, different concentrations of exogenous benzoic acid and cinnamic acid significantly reduced the activity of CAT and had a concentration effect, and the enzyme activity is the highest at the peak of the disease (Guo et al., 2020). Similar results were obtained in the strawberry planting system. Qi et al. (2016) found that the continuous addition of *p*-hydroxybenzoic acid, an autotoxic substance in strawberry continuous cropping, and inoculation with *F. oxysporum* can significantly reduce the SOD activity of strawberry plants and increase the peroxidation of root membranes, which promotes the occurrence of strawberry wilt. In this study, the exogenous addition of benzoic acid and cinnamic acid caused an oxidative explosion in faba beans, producing a large amount of H_2_O_2_ and O_2_^−^, and high concentrations of benzoic acid and cinnamic acid significantly reduced the activities of SOD and CAT. That destroys the antioxidant system of faba beans, reduces the resistance of faba beans to biological stress, and destroys the faba bean cells, eventually resulting in the death of the plant.

We also found in pot experiments that in each treatment, the faba bean–wheat intercropping significantly reduced the contents of H_2_O_2_ and O_2_^−^ compared with monocropping and effectively reduced the oxidative damage caused by benzoic acid and cinnamic acid to faba beans. Similar results were obtained in the intercropping system of rice and watermelon. Research by Ren et al. (2016) showed that rice and watermelon intercropping can significantly reduce the content of ROS, reduce oxidative damage, and effectively alleviate the occurrence of watermelon wilt, which is consistent with our results. The decrease in H_2_O_2_ and O_2_^−^ is the effect of plant antioxidant enzymes SOD and CAT. In each treatment, compared with monocropping, faba bean–wheat intercropping significantly increased the enzyme activity and expression of the CAT and SOD genes. This shows that the faba bean–wheat intercropping system can reduce the autotoxic stress of benzoic acid and cinnamic acid on faba beans by improving the activity of the faba bean antioxidant system. This is consistent with the research of Cheng et al. (2020), using garlic and tomato intercropping to significantly improve the activity of tomato defense enzymes, enhance tomato resistance, and reduce the occurrence of diseases. In addition, compared with monoculture, faba bean–wheat intercropping effectively reduced the plasmolysis of root cells, protected the integrity of root cells, and alleviated the damage of benzoic acid and cinnamic acid on the root cells of faba bean. This prevents the H_2_O_2_ in ROS from passing through the cell membrane and invading the cell. The intercropping of Bahia grass (*Paspalum notatum*) and olive (*Olea europaea*) significantly increased the cell membrane stability index of the olive and improved its ability to resist stress (Yang et al., 2016). This is similar to our results.

## Conclusion

The experiment proved that after inoculation with FOF, faba beans try to mobilize the antioxidant system and increase the gene expression of CAT and SOD to resist the infection of FOF. However, in the end, the antioxidant system is still destroyed, and the antioxidant enzyme activity is significantly reduced. The high concentration of benzoic acid and cinnamic acid could damage the antioxidant system, increase the degree of peroxidation in the root system of faba bean, destroy the structure of the cell membrane, and promote the occurrence of *Fusarium* wilt of faba bean. The faba bean–wheat intercropping can effectively alleviate the autotoxicity of benzoic acid and cinnamic acid and reduce the occurrence of faba bean *Fusarium* wilt.

## Data availability statement

The original contributions presented in the study are included in the article/**Supplementary Materials**. Further inquiries can be directed to the corresponding author.

## Author contributions

YZ and YG conceived the original screening and research plans, finished writing this thesis. YL and WY supervised the experiments, agreed to serve as the author responsible for contact and ensures communication. YD provided technical assistance to YZ. YZ designed the experiments. YG analyzed the data. All authors contributed to the article and approved the submitted version.

## Funding

This work was supported by the Natural Science Foundation of China (31860596).

## Conflict of interest

The authors declare that the research was conducted in the absence of any commercial or financial relationships that could be construed as a potential conflict of interest.

## Publisher’s note

All claims expressed in this article are solely those of the authors and do not necessarily represent those of their affiliated organizations, or those of the publisher, the editors and the reviewers. Any product that may be evaluated in this article, or claim that may be made by its manufacturer, is not guaranteed or endorsed by the publisher.

## References

[B1] Alexandratos N. Bruinsma J. (2012). World agriculture towards 2030/2050: the 2012 revision (Agricultural Develop5ment Economics Division. Agriculture Organization of the United Nations: ESA Working Paper No.12-03). doi: 10.22004/ag.econ.288998

[B2] Beckman C. H. (1987). “The nature of wilt diseases of plants,” in Quarterly review of biology (St. Paul, MN: American Phytopathological Society Press). doi: 10.2307/2831038

[B3] Bonanomi G. Chiurazzi M. Caporaso S. Sorbo G. D. Moschetti G. Felice S. (2008). Soil solarization with biodegradable materials and its impact on soil microbial communities. Soil Biol. Biochem. 40, 1989–1998. doi: 10.1016/j.soilbio.2008.02.009

[B4] Cebolla B. Busto J. Ferrer A. Miguel A. Maroto J. V. (2000). Methyl bromide alternatives on horticultural crops. Acta Hortic. 532, 237–242. doi: 10.17660/ActaHortic.2000.532.32

[B5] Cheng F. Ali M. Liu C. Deng R. Cheng Z. (2020). Garlic allelochemical diallyl disulfide alleviates autotoxicity in the root exudates caused by long-term continuous cropping of tomato. J. Agric. Food Chem. 68 (42), 11684–11693. doi: 10.1021/acs.jafc.0c03894 32991155

[B6] De Borba M. C. Garcés-Fiallos F. R. Stadnik M. J. (2017). Reactions of black bean seedlings and adult plants to infection by fusarium oxysporum f. sp. phaseoli. Crop Prot. 96, 221–227. doi: 10.1016/j.cropro.2017.02.019

[B7] Dong Y. Dong K. Tang L. Zheng Y. Yang Z. Xiao J. (2013). Effect of wheat and broad bean intercropping on the functional diversity of broad bean rhizosphere microbial community and its relationship with the occurrence of broad bean fusarium. Chin. J. Ecol. J. 33 (23), 7445–7454.

[B8] Fravel D. R. Deahl K. L. Stommel J. R. (2005). Compatibility of the biocontrol fungus fusarium oxysporum strain CS-20 with selected fungicides. Biol. Control 34, 165–169. doi: 10.1016/j.biocontrol.2005.04.007

[B9] Gebru H. (2015). Effect of planting patterns in tomato (Lycopersicon esculentum Mill) and maize (Zea mays L.) intercropping on growth, yield and yield traits of the crops in wolaita Zone, Southern Ethiopia. J. Biol. Agric. Healthcare. 5(1), 39–49.

[B10] Gill S. S. Tuteja N. (2010). Reactive oxygen species and antioxidant machinery in abiotic stress tolerance in crop plants. Plant Physiol. Biochem. 48, 909–930. doi: 10.1016/j.plaphy.2010.08.016 20870416

[B11] Gómez-Rodrıguez O. Zavaleta-Mejıa E. Gonzalez-Hernandez V. A. Livera-Munoz M. Cárdenas-Soriano E. (2003). Allelopathy and microclimatic modification of intercropping with marigold on tomato early blight disease development. Field Crops Res. 83 (1), 27–34. doi: 10.1016/S0378-4290(03)00053-4

[B12] Guo Y. Lv J. Zhao Q. Dong Y. Dong K. (2020). Cinnamic acid increased the incidence of fusarium wilt by increasing the pathogenicity of fusarium oxysporum and reducing the physiological and biochemical resistance of faba bean, which was alleviated by intercropping with wheat. Front. Plant Sci. 11. doi: 10.3389/fpls.2020.608389 PMC7767866 33381139

[B13] Gutierrez N. María J. Giménez Palomino C. Avila C. M. (2011). Assessment of candidate reference genes for expression studies in vicia faba l. by real-time quantitative pcr. Mol. Breed. 28 (1), 13–24. doi: 10.1007/s11032-010-9456-7

[B14] Hao W. Y. Ren L. X. Ran W. Shen Q. R. (2010). Allelopathic effects of root exudates from watermelon and rice plants on fusarium oxysporum f.sp. niveum. Plant Soil 336, 485–497. doi: 10.1007/s11104-010-0505-0

[B15] Hartman K. van der Heijden M. G. Wittwer R. A. Banerjee S. Walser J. C. Schlaeppi K. (2018). Cropping practices manipulate abundance patterns of root and soil microbiome members paving the way to smartfarming. Microbiome 6 (1), 1–14. doi: 10.1186/s40168-017-0389-9 29338764 PMC5771023

[B16] Hasanuzzaman M. Bhuyan M. Parvin K. Bhuiyan T. F. Fujita M. (2020a). Regulation of ros metabolism in plants under environmental stress: a review of recent experimental evidence. Int. J. Mol. Sci. 21 (22), 8695. doi: 10.3390/ijms21228695 PMC7698618 33218014

[B17] Hasanuzzaman M. Bhuyan M. Zulfiqar F. Raza A. Mohsin S. M. Mahmud J. A.. (2020b). Reactive oxygen species and antioxidant defense in plants under abiotic stress: revisiting the crucial role of a universal defense regulator. Antioxidants 9 (8), 681. doi: 10.3390/antiox9080681 PMC7465626 32751256

[B18] Jin X. Wang Z. Wu F. Li X. Zhou X. (2022). Litter mixing alters microbial decomposer community to accelerate tomato root litter decomposition. Microbiol. Spectr. e00186-22. doi: 10.1128/spectrum.00186-22 PMC9241821 35604181

[B19] Liang G. Q. Sun J. W. Zhou W. Wang X. B. (2011). Effects of calcium on activities and gene expressions of superoxide dismutase and catalase in apple (malus pumila mill.) fruits. Plant Nutr. Fertilizer Sci. 17, 438–444. doi: 10.11674/zwyf.2011.9502

[B20] Lin W. X. Kim K. U. Shin D. H. (2000). Rice allelopathic potential and its modes of action on barnyardgrass (Echinochloa crusgalli).Chin. J. Allelopathy J. 7, 215–224.

[B21] Liu H. Gao Y. Gao C. Liu S. Zhang J. Chen G. (2019). Study of the physiological mechanism of delaying cucumber senescence by wheat intercropping pattern. J. Plant Physiol. 2. doi: 10.1016/j.jplph.2019.02.003 30818185

[B22] Liu J. Li X. Jia Z. Zhang T. Wang X. (2016). Effect of benzoic acid on soil microbial communities associated with soilborne peanut diseases. Appl. Soil Ecol. 110, 34–42. doi: 10.1016/j.apsoil.2016.11.001

[B23] Li X. G. Zhang T. L. Wang X. X. Hua K. Zhao L. Han Z. M. (2013). The composition of root exudates from two different resistant peanut cultivars and their effects on the growth of soil-borne pathogen. Int. J. Biol. Sci. 9 (2), 164. doi: 10.7150/ijbs.5579 23412138 PMC3572399

[B24] Lopes T. Hatt S. Xu Q. Chen J. Yong L. Francis H. (2016). Wheat (triticum aestivum l.)-based intercropping systems for biological pest control. Pest Manage. Sci. 72. doi: 10.1002/ps.4332 27271821

[B25] Lv H. Cao H. Nawaz M. A. Sohail H. Huang Y. Cheng F.. (2018). Wheat intercropping enhances the resistance of watermelon to fusarium wilt. Front. Plant Sci. 9. doi: 10.3389/fpls.2018.00696 PMC5980984 29887873

[B26] Niño-Sánchez J. Tello V. Casado-del Castillo V. Thon M. R. Benito E. P. Díaz-Mínguez J. M. (2015). Gene expression patterns and dynamics of the colonization of common bean (Phaseolus vulgaris l.) by highly virulent and weakly virulent strains of fusarium oxysporum. Front. Microbiol. 6, 234. doi: 10.3389/fmicb.2015.00234 25883592 PMC4383042

[B27] Qi Y. Jin J. Chang N. Zhang X. Yin B. Zhen W. (2016). Promoting effect of p-hydroxybenzoic acid on the occurrence of strawberry wilt. Chin. J.China Plant Prot. Guide 6, 5–10.

[B28] Ren L. Huo H. Zhang F. Hao W. Xiao L. Dong C. (2016). The component difference between rice and watermelon root exudates which lead to their ecological roles on pathogenic fungus and watermelon defense. Plant Signaling Behav. 2, e1187357. doi: 10.1080/15592324.2016.1187357 PMC4977455 27217091

[B29] Ren L. X. Su S. M. Yang X. M. Xu Y. C. Huang Q. W. Shen Q. R. (2008). Intercropping with aerobic rice suppressed fusarium wilt in watermelon. Soil Biol. Biochem. 40, 834–844. doi: 10.1016/j.soilbio.2007.11.003

[B30] Siddiqui M. H. Al-Khaishany M. Y. Al-Qutami M. A. Al-Whaibi M. H. Bukhari N. A. (2015). Response of different genotypes of faba bean plant to drought stress. Int. J. Mol. Sci. 16 (5), 10214–10227. doi: 10.3390/ijms160510214 25950766 PMC4463642

[B31] Singh N. B. (2015). Alleviation of allelopathic stress of benzoic acid by indole acetic acid in solanum lycopersicum. Scientia horticulturae. 192, 211–217. doi: 10.1016/j.scienta.2015.06.013

[B32] Su F. Y. Yan H. Z. Yao S. Li Y. Z. Jing Q. Y. (2006). Cinnamic acid causes oxidative stress in cucumber roots, and promotes incidence of fusarium wilt. Environ. Exp. Bot. 56, 255–262. doi: 10.1016/j.envexpbot.2005.02.010

[B33] Tamire Z. Chemeda F. (2007). Association of white rot (sclerotium cepivorum) of garlic with environmental factors and cultural practices in the north shewa highlands of ethiopia. Crop Prot. 26, 1566–1573. doi: 10.1016/j.cropro.2007.01.007

[B34] Tanha A. Golzardi F. Mostafavi K. (2017). Seed priming to overcome autotoxicity of alfalfa (Medicago sativa). World J. Environ. Biosci. 6, 1–5.

[B35] Tan G. Liu Y. Peng S. Yin H. Zhou Z. (2021). Soil potentials to resist continuous cropping obstacle: three field cases. Environ. Res. 6, 111319. doi: 10.1016/j.envres.2021.111319 34052246

[B36] Tilman D. Balzer C. Hill J. Befort B. L. (2011). Global food demand and the sustainable intensification of agriculture. Proc. Natl. Acad. Sci. United States America 108, 20260–20264. doi: 10.1073/pnas.111643710 PMC3250154 22106295

[B37] Wang X. Q. Du G. D. Lu X. F. Ma H. Y. Lyu D. G. Zhang H.. (2019). Characteristics of mitochondrial membrane functions and antioxidant enzyme activities in strawberry roots under exogenous phenolic acid stress. Scientia horticulturae. 248, 89–97. doi: 10.1016/j.scienta.2018.12.051

[B38] Wang M. Wu C. Cheng Z. Meng H. (2015). Growth and physiological changes in continuously cropped eggplant (solanum melongena l.) upon relay intercropping with garlic (allium sativum l.). Front. Plant Sci. 6. doi: 10.3389/fpls.2015.00262 PMC4408842 25964788

[B39] Wu H. Zhou X. Shi X. Liu Y. Wang M. Shang X.. (2014). *In vitro* responses of fusarium oxysporum f.sp.niveum to phenolic acids in decaying watermelon tissues. Phytochem. Lett. 8, 171–178. doi: 10.1016/j.phytol.2013.08.013

[B40] Xiong W. Zhao Q. Zhao J. Xun W. Li R. Zhang R. (2015). Different continuous cropping spans significantly affect microbial community membership and structure in a vanilla-grown soil as revealed by deep pyrosequencing. Microbial Ecol. 70(1), 209–218. doi: 10.1007/s00248-014-0516-0 25391237

[B41] Yang H. Li Z. Liang S. Jiao J. Li C. (2016). The effect of intercropping bahia grass on the rhizosphere microenvironment and drought resistance physiology of olea europaea. Chin. J. Chin. J. Appl. Environ. Biol. 22, 7.

[B42] Yang M. Youcong C. Guo C. Liao J. Xu Y. Mei X. (2018). Panax notoginseng root cell death caused by the autotoxic ginsenoside rg1 is due to over-accumulation of ros, as revealed by transcriptomic and cellular approaches. Front. Plant Sci. 9. doi: 10.3389/fpls.2018.00264 PMC5836058 29541087

[B43] Zhang Z. Wang J. Chen L. Fan J. Han X. Wang G. (2018). Cloning and expression analysis of the muskmelon autotoxic stress response gene cmfe-sod. J. Fruit Sci. 35, 9. doi: 10.13925/j.cnki.gsxb.20180132

[B44] Zhou X. Liu J. Wu F. (2017). Soil microbial communities in cucumber monoculture and rotation systems and their feedback effects on cucumber seedling growth. Plant Soil 415 (1), 507–520. doi: 10.1007/s11104-017-3181-5

[B45] Zhou X. Zhang J. Pan D. Ge X. Jin X. Chen S.. (2018). P-coumaric can alter the composition of cucumber rhizosphere microbial communities and induce negative plant-microbial interactions. Biol. Fertility Soils 54 (3), 363–372. doi: 10.1007/s00374-018-1265-x

[B46] Zulfiqar F. Akram N. A. Ashraf M. (2020). Osmoprotection in plants under abiotic stresses:New insights into a classical phenomenon. Planta. 251(1), 1–17. doi: 10.1007/S00425-019-03293-1 31776765

[B47] Zulfiqar F. Ashraf M. (2021a). Bioregulators: unlocking their potential role in regulation of the plant oxidative defense system. Plant Mol. Biol. 105(1), 11–41. doi: 10.1007/s11103-020-01077-w 32990920

[B48] Zulfiqar F. Ma B. (2021b). Antioxidants as modulators of arsenic-induced oxidative stress tolerance in plants: an overview. J. Hazardous Materials 127891. doi: 10.1016/j.jhazmat.2021.127891 34848065

