# Supplementary material for: Intercropping of wheat alleviates the adverse effects of phenolic acids on faba bean
Source: Front Plant Sci. 2022 Oct 17;13:997768. doi: 10.3389/fpls.2022.997768 (PMC9618816; doi:10.3389/fpls.2022.997768)
Supplement: Supplementary file 1 [file DataSheet_1.doc]

**
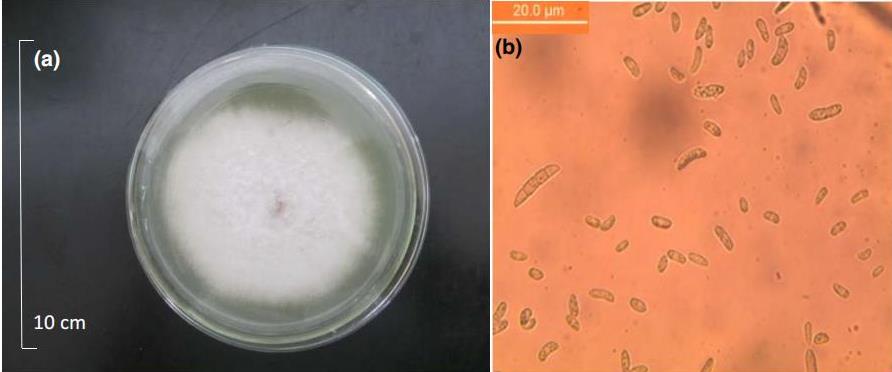
**

**Figure S1** Fusarium oxysporum isolated from the root system of faba bean in the field. (a) F. oxysporum cultured on a Petri dish; (b) F. oxysporum spores observed with a microscope**
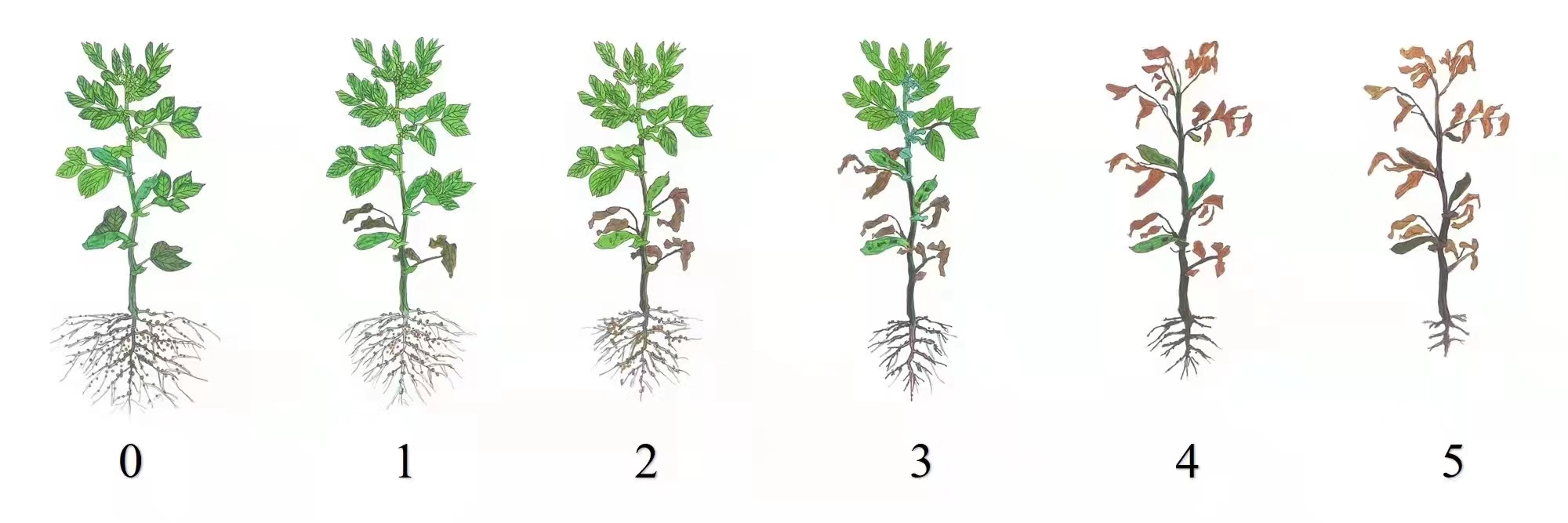
**

**Figure S2.**Disease rating scores for faba bean Fusarium wilt. From left to right, scores of 0–5

**
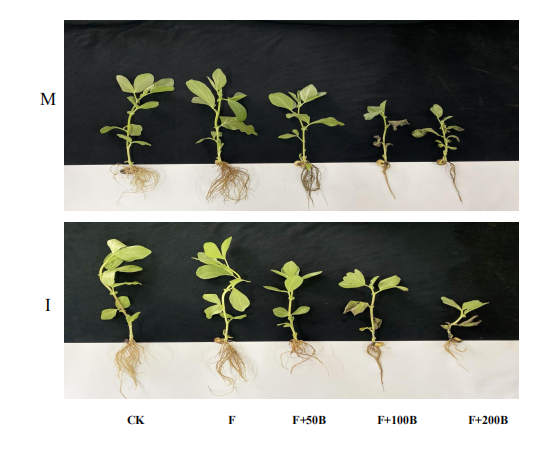

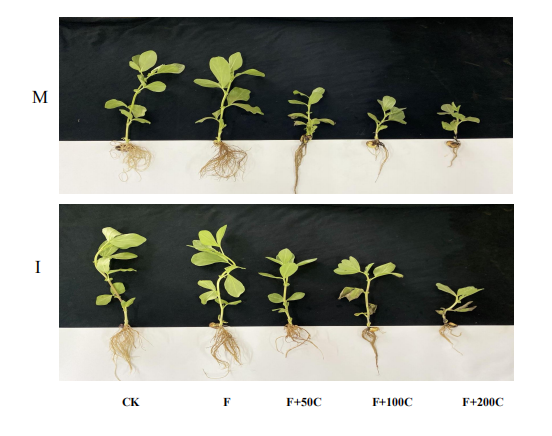
**

**Figure S3.**Phenotypic symptomatic pictures of faba bean (*Vicia faba*)with different concentrations of exogenous benzoic acid and cinnamic acid under different cropping modes.M: faba bean monocropping; I: wheat-faba bean intercropping


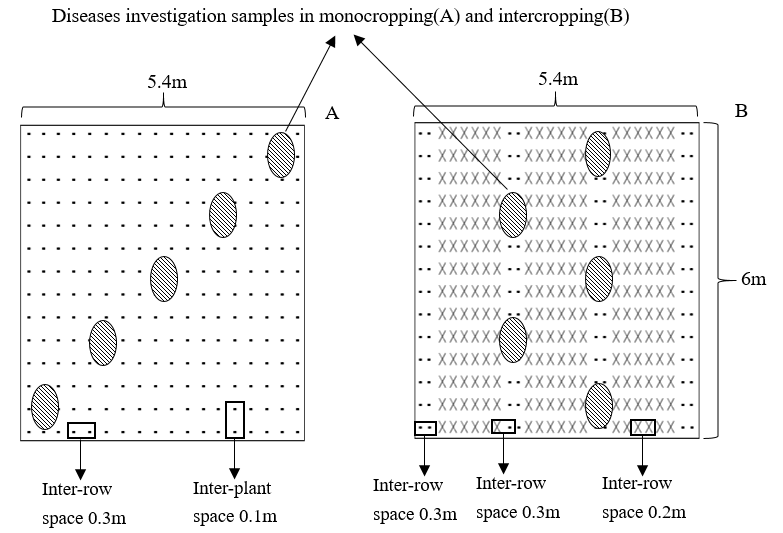


**Figure S4**. Diagram of the planting patterns in the field experiments: (A) the monocropping faba bean plot, (B) the intercropping plot of faba bean with wheat. - , faba bean; ×, wheat; shaded ovals representative sampling locations.

**RNA extraction**

Homogenate processing: 50-100 mg of plant tissue was quickly ground into powder in liquid nitrogen, and 500 µL of RLS Buffer was used ensuring that the buffer contained ß-mercaptoethanol before use. The sample was immediately mixed by vortexing. The sample was then centrifuged at 13,400×g for 2 min at 4°C. The supernatant was transferred to a filtration column (Spin Columns FS), loaded into the collection tube and centrifuged at 12,000 rpm at 4°C for 1 min. The supernatant was carefully drained from the collection tube and transferred to a new RNase-free centrifuge tube. A volume of 0.5 times the supernatant volume of absolute ethanol was added and mixed well. The resulting solution and precipitate were transferred into the adsorption column (Spin Columns RM) that had been loaded into the collection tube. All the solution should be added to the adsorption column and transferred in twice. Next, the solution was centrifuged at 12,000 rpm at 4°C for 1 minute, and the waste liquid was discarded. The adsorption column was placed back into the collection tube. A volume of 350 µL buffer RW1 was added to the adsorption column RM and centrifuged at 12,000 rpm at 4°C for 1 min. The waste liquid was discarded, and the adsorption column placed back into the collection tube. The DNase I mixture was prepared by taking 52 µL RNase-Free Water, adding 8 µL of 10×Reaction Buffer and 20 µL of DNase I (1 U/µL) to it, mixing well, and preparing a final volume of 80 µL reaction solution. A volume of 80 µL of the DNase I mixture was added directly to the adsorption column and incubated at 20-30°C for 15 minutes. A volume of 350 µL of Buffer RW1 was added to the adsorption column RM and centrifuged at 12,000 rpm at 4°C for 1 min. The waste liquid was discarded, and the adsorption column was placed back into the collection tube. A volume of 500 µL Buffer RW2 was added to the adsorption column RM after checking whether ethanol was added before use and centrifuged at 12,000 rpm at 4°C for 1 min. The waste liquid was discarded and the adsorption column was placed back into the collection tube. Previous step was repeated and centrifuged at 12,000 rpm at 4°C for 2 minutes. The samples should be stored in sub-packages. Two to three sub-packages of qualified quality should be stored at -80°C for later use.

**cDNA preparation**

The RNA was diluted to 500 ng·µL^-1^, and 1 ng of RNA was added for reverse transcription. The reverse transcription operation was performed on ice, and all the equipment had been treated with denuclease.

The method and steps used VHiScript II Q RT SuperMix for qPCR (+gDNA wiper) reagent as an example.

**Experimental process:**

(1) The genomic DNA was removed and the following mixed solution was configured in a 1.5 mL EP tube.

Table S1. Preparation of genomic DNA removal mixture.

| Component | Volume |
| --- | --- |
| 4 × gDNA wiper Mix | 2 µL |
| Templet RNA | Total RNA: 1 µg |
| RNase free ddH_2_O | to 8 µL |

After adding the system, gently blow the mixture with a pipette. Treat at 42℃ for 2 min.

1. The reverse transcription reaction system was prepared (5 HiScript II qRT SuperMix II) and directly added into (1).

**Table S2. Reverse transcription reaction system**

| Component | Volume | |
| --- | --- | --- |
| 5 × HiScript II qRT SuperMix II | | 2 µL |
| Reaction liquid in (1) | | 8 µL |

The mixture was gently mixed with a pipette.

(3) Reverse transcription (RT-PCR) reaction.

The PCR products can be used immediately for quantitative PCR (qPCR) reactions or stored at -20°C and used within six months.

Table S3. Reverse transcription reaction PCR program

| Temperature | Time |
| --- | --- |
| 50 ℃ | 15 min |
| 85 ℃ | 5 sec |

Table S4. qPCR reaction conditions

| Stage | Temperature | Time | Cycle number |
| --- | --- | --- | --- |
| Predegeneration^1^ | 95℃ | 30 sec | 1 |
| Circular reaction^2^ | 95℃ | 10 sec | 40 |
|  | 60℃ | 30 sec |  |
| Solubility curve^3^ | 95℃ | 15 sec | 1 |
|  | 60℃ | 60 sec |  |
|  | 95℃ | 15 sec |  |

Fluorescence quantitative PCR was used to detect the expression of 6 defense genes *VfCAT、VfSOD* in faba bean roots.

Table S5. Defense gene primer sequence

| Gene | Sequence |
| --- | --- |
| *Vf reference gene*  (CYP2) | TGCCGATGTCACTCCCAGAA |
|  | CAGCGAACTTGGAACCGTAGA |
| *Vf reference gene*  (EFL1A) | GTGAAGCCCGGTATGCTTGT |
|  | CTTGAGATCCTTGACTGCAACATT |
| *VfSOD* | CTGCCGCCAAGAAAGCC |
|  | GGTCCTGTTGAGATACACCCATT |
| *VfCAT* | GATTTTGACCCACTTGATGTAACC |
|  | AGGCACGATAATGGCAGGAC |

the results of altered soil microbiome, changes in carbon resource quality in the soil, more rapid degradation of autotoxins
